# A malaria parasite subtilisin propeptide-like protein is a potent inhibitor of the egress protease SUB1

**DOI:** 10.1042/BCJ20190918

**Published:** 2020-01-31

**Authors:** Sarah J. Tarr, Chrislaine Withers-Martinez, Helen R. Flynn, Ambrosius P. Snijders, Laura Masino, Konstantinos Koussis, David J. Conway, Michael J. Blackman

**Affiliations:** 1 Faculty of Infectious and Tropical Diseases, London School of Hygiene and Tropical Medicine, London WC1E 7HT, U.K.; 2 Malaria Biochemistry Laboratory, The Francis Crick Institute, 1 Midland Rd, London NW1 1AT, U.K.; 3 Protein Analysis and Proteomics Platform, The Francis Crick Institute, 1 Midland Rd, London NW1 1AT, U.K.; 4 Structural Biology Science Technology Platform, The Francis Crick Institute, 1 Midland Rd, London NW1 1AT, U.K.

**Keywords:** parasitophorous vacuole, *Plasmodium falciparum*, propeptide, subtilase

## Abstract

Subtilisin-like serine peptidases (subtilases) play important roles in the life cycle of many organisms, including the protozoan parasites that are the causative agent of malaria, *Plasmodium* spp. As with other peptidases, subtilase proteolytic activity has to be tightly regulated in order to prevent potentially deleterious uncontrolled protein degradation. Maturation of most subtilases requires the presence of an N-terminal propeptide that facilitates folding of the catalytic domain. Following its proteolytic cleavage, the propeptide acts as a transient, tightly bound inhibitor until its eventual complete removal to generate active protease. Here we report the identification of a stand-alone malaria parasite propeptide-like protein, called SUB1-ProM, encoded by a conserved gene that lies in a highly syntenic locus adjacent to three of the four subtilisin-like genes in the *Plasmodium* genome. Template-based modelling and *ab initio* structure prediction showed that the SUB1-ProM core structure is most similar to the X-ray crystal structure of the propeptide of SUB1, an essential parasite subtilase that is discharged into the parasitophorous vacuole (PV) to trigger parasite release (egress) from infected host cells. Recombinant *Plasmodium falciparum* SUB1-ProM was found to be a fast-binding, potent inhibitor of *P. falciparum* SUB1, but not of the only other essential blood-stage parasite subtilase, SUB2, or of other proteases examined. Mass-spectrometry and immunofluorescence showed that SUB1-ProM is expressed in the PV of blood stage *P. falciparum*, where it may act as an endogenous inhibitor to regulate SUB1 activity in the parasite.

## Introduction

Proteolytic enzymes perform numerous regulatory and degradative roles across the kingdoms of life. A universal requirement for the expression of these potentially highly damaging enzymes in all organisms is tight control of their proteolytic activity in time and space. This is achieved in a multitude of ways, ranging from the stringent regulation of protease maturation through to the strategic expression of endogenous regulatory molecules that act *in trans* to positively or negatively modulate protease activity. Members of the second largest family of serine peptidases, the subtilisin-like serine proteases (clan SB family S8, or subtilases) are typically expressed as enzymatically inactive zymogens which, in the case of secreted subtilases, comprise a single polypeptide that minimally possess a (usually) N-terminal prodomain, or propeptide, appended to the catalytic domain. The propeptide acts as an ‘intramolecular chaperone’, being essential for correct folding of the catalytic domain. In addition, maturation of the protease is generally associated with proteolytic cleavage of the propeptide segment, which continues to act as a temporary high-affinity bound inhibitor until its complete removal by further proteolysis and/or a change in environmental conditions. Removal of the propeptide generates the free, enzymatically active protease (for an excellent review of subtilisin maturation, see [1]). Subtilisin propeptides are usually therefore potent, competitive inhibitors of their cognate enzymes (with *K_i_* values typically in the low nM range) and in some instances have also been shown to inhibit heterologous subtilisins too (e.g. [2]).

In acknowledgement of the inhibitory potency of subtilisin propeptides, the MEROPS database of peptidases and peptidase inhibitors (see https://www.ebi.ac.uk/merops/) includes several bona fide subtilisin propeptides within its I9 family (clan JC) of inhibitors. The I9 family also includes three well-characterised proteins that, despite lacking significant sequence similarity, resemble ‘stand-alone’ subtilisin propeptides, being small gene products in their own right that closely mimic the propeptide fold but that are not derived from larger enzyme precursors. All three proteins (*Pleurotus ostreatus* peptidase A inhibitor 1, *Saccharomyces cerevisiae* peptidase B inhibitor and *Arabidopsis thaliana* subtilisin propeptide like inhibitor 1), display typically potent inhibition of one or more subtilisin-like proteases [3–6]. A further recently characterised example of a subtilisin propeptide-like mimic is microneme protein 5 (TgMIC5; [7]) of the parasitic protist *Toxoplasma gondii*, which regulates the activity of a subtilase that ‘trims’ surface proteins involved in motility of the parasite [8]. The existence of such stand-alone propeptide-like proteins may reflect a process of convergent evolution [9] in which the subtilisin propeptide fold has been adopted to carry out regulatory protease inhibitory functions independent of any chaperone activity.

Malaria is caused by an obligate intracellular protozoan parasite that shares its life cycle between an Anopheline mosquito vector and a vertebrate host. In the latter, the parasite undergoes a transient amplification step in the liver before initiating its asexual blood-stage life cycle, which comprises repeated cycles of invasion into red blood cells (RBC), intracellular replication to form a multinucleated form called a schizont, then formation and egress of invasive merozoites with concomitant lytic destruction of the host RBC. The malarial genome encodes just four secreted subtilisin-like proteins, each of which has orthologues in all *Plasmodium* species examined. Three of these (termed SUB1, SUB2 and SUB3) are expressed as enzymatically active proteases [10–16], whereas the fourth (PIMMS2 or SOPT) has unusual features including a non-canonical catalytic triad that suggest that it likely lacks enzyme activity [17,18]. The best-characterised member of the *Plasmodium* subtilase family is SUB1, which is initially stored in a set of secretory organelles of the developing merozoite called exonemes [19]. Minutes before egress of mature merozoites from the infected RBC, SUB1 is discharged under the control of a cGMP-dependent signal into the lumen of the parasitophorous vacuole (PV), the intraerythrocytic compartment within which the parasite replicates. There, SUB1 precisely cleaves a number of resident soluble and merozoite surface proteins [19–21]. Gene disruption and mutagenesis studies have revealed essential roles for *Plasmodium falciparum* SUB1 (PfSUB1) and its protein substrates in asexual blood-stage parasite egress [22–24]. SUB1 is also required for egress of the liver stage merozoites that initiate the blood-stage lifecycle [25,26], identifying this subtilase as an important player in parasite replication and virulence.

Here we identify a stand-alone subtilisin propeptide-like protein that is probably expressed in the PV of asexual blood stages of the malaria parasite and that is a potent and selective inhibitor of SUB1.

## Materials and methods

### Antibodies and reagents

Rabbit polyclonal antibodies specific for the *P. falciparum* merozoite surface protein MSP4 (MRA-319, kind gifts of Ross Coppel, Monash University, Australia) were obtained through BEI Resources, National Institute of Allergy and Infectious Diseases, National Institutes of Health, Rockville, MD 20852, U.S.A., and used in immunofluorescence analysis (IFA) at a dilution of 1:200. A rabbit polyclonal antiserum against SERA5 has been described previously [27], and was used at a 1 : 2000 dilution. Monoclonal antibodies X509 [28] and 89.1 [29], specific for *P. falciparum* MSP1, have also been previously described. For generation of antibodies to SUB1-ProM, five CD1 mice were immunised with purified recombinant glutathione S-transferase (GST)-SUB1-ProM (50 µg protein per immunisation), boosting at days 14, 28 and 56, with a terminal bleed on day 87. Serum was collected after storage of the blood overnight at 4°C. Reactivity of individual antisera was determined by IFA of fixed *P. falciparum* schizonts. Four sera showing reactivity were pooled in equal proportions and subsequently used in IFA at a final dilution of 1 : 50. Production and purification of chymotrypsin-treated recombinant *P. falciparum* SUB1 (rPfSUB1) and design of the fluorogenic dodecapeptide PfSUB1 substrate SERA4st1F-6R12, based on a PfSUB1 cleavage site within the endogenous protein substrate SERA4, have been described previously [14,30]. Rapamycin and subtilisin Carlsberg (SUBC, *Bacillus licheniformis*) were from Sigma, and pronase (from *Streptomyces griseus*) was from Boehringer.

### Parasites and IFA

Wild type *P. falciparum* clone 3D7 and the transgenic *P. falciparum* clones SUB1HA3:*loxP* [24] and B11 [31] were routinely maintained and synchronised using standard methods [32] in cultures containing human erythrocytes at a 2.5% haematocrit in RPMI 1640 medium containing 0.5% (w/v) Albumax II (Life Technologies, U.K.) in an atmosphere of 5% O_2_, 5% CO_2_, and 90% N_2_. For analysis by IFA, mature, Percoll-enriched schizonts were resuspended with uninfected erythrocytes at 50% haematocrit to a final parasitaemia of ∼1%. Thin films were air dried then fixed at room temperature in 4% paraformaldehyde/0.0075% glutaraldehyde in phosphate-buffered saline (PBS) for 30 min. Fixative was exchanged for 0.1% Triton X-100 in PBS for 10 min to permeabilize the cells, then slides were blocked overnight in 3% (w/v) BSA in PBS at 4°C. Blocked slides were rinsed in PBS for 5 min, then excess PBS removed and discrete regions drawn on to the smear with a liquid blocker pen (Thermo Fisher Scientific). These regions were incubated with primary antibodies overnight at 4°C, then washed three times in PBS with shaking, followed by incubation for 2 h in the dark with combinations of secondary antibodies. These were as appropriate: goat anti-mouse Alexa Fluor 488 (A-11029; Thermo Fisher Scientific; used at 1 : 1000); and goat anti-rabbit Alexa Fluor 594 (A-11037; Thermo Fisher Scientific; used at 1 : 1000). Slides were washed three times in PBS and sealed under coverslips with Fluoroshield mounting fluid containing 4,6-diamidino-2-phenylindol (DAPI) for nuclear staining (Sigma). Samples were imaged using a Zeiss Axiovision wide field fluorescence microscope and Volocity deconvolution software.

### Bioinformatics analysis

The *Plasmodium* genomic resource PlasmoDB (http://plasmodb.org/plasmo/) was used to identify and select PF3D7_0507400 (PfSUB1-ProM) orthologues from the available synteny list: these were PGSY75_0507400, *P. gaboni*; PFIT_0507400, *P. falciparum*; PRCDC_0506600, *P. reichenowi*; PCHAS_110670, *P. chabaudi chabaudi*; PY17X_1108100, *P. yoelii*; PBANCA_1107000, *P. berghei*; PKNH_1026200, *P. knowlesi*; PKNOH_S07464200, *P. knowlesi*; PVX_097930, *P. vivax*; PcyM_1026600, *P. cynomolgi*; PCYB_103420, *P. cynomolgi*; PGAL8A_00028500, *P. gallinaceum*; PRELSG_1025100, *P. relictum*; PocGH01_10034100, *P. ovale curtisi*. The Clustal Omega server (https://www.ebi.ac.uk/Tools/msa/clustalo/, EMBL-EBI Services) was used to align all the predicted primary amino acid sequences and to provide an associated identity matrix. The resulting alignment was fed to the utility rendering program ESPrit3 (http://espript.ibcp.fr/ESPript/ESPript/) together with the PDB file from the Modeller SUB1-ProM output model in order to visualise secondary structure information from the model together with the alignment sequences.

### Protein structure prediction

Modeller v9.20 (https://salilab.org/modeller/release.html) was used in conjunction with the UCL psipred Profile Based Fold Recognition routine pGenTHREADER (http://bioinf.cs.ucl.ac.uk/psipred/) to generate a predicted structure of SUB1-ProM based on the best-identified template (PDB ID: 4LVN). The model with the lowest molpdf score and the best geometry from the Ramachandran plot statistics (preferred 91%, allowed 2.26%, outlier 6.74%) was chosen out of the five models generated by Modeller. I-Tasser (Iterative Threading ASSEmbly Refinement; https://zhanglab.ccmb.med.umich.edu/I-TASSER/), which uses a meta-threading method (Local Meta-Threader Server) for template-based protein structure prediction, was also used to generate a SUB1-ProM model, the co-ordinates of which were fed to the PDBeFold server (www.ebi.ac.uk/msd-srv/ssm/) to search for a similar fold in the PDB. The same strategies were used to model the *Plasmodium yoelii* orthologue PY17X_1108100.

The Robetta protein structure prediction server (http://robetta.bakerlab.org) and the QUARK server (https://zhanglab.ccmb.med.umich.edu/QUARK/) were used for *ab initio* modelling. Visualisation, manipulation and images of the models were made within the PyMOL Open-Source Molecular Graphic System (https://pymol.org/2/).

### Recombinant expression of GST-rSUB1-ProM and purification of rSUB1-ProM

A synthetic gene corresponding to residues 28–153 of PF3D7_0507400 (SUB1-ProM) was cloned into the GST-tag expression vector pGEX-6P-1 (GE Healthcare) with an added human rhinovirus 3C protease cleavage site (kindly provided by Andrew Osborne, University College London) and transformed into BL21(R2) *E. coli* cells cultured in the presence of ampicillin (100 µg/ml) and chloramphenicol (34 µg/ml). A freshly picked colony was used to grow a starter culture from which 1 ml was seeded into 1 litre Luria broth supplemented with ampicillin (100 µg/ml) in a 5 litre flask. Protein production was induced with 1 mM IPTG when the OD600 of the culture reached 0.4–0.6. The cells were harvested by centrifugation after overnight growth at 37°C. Pellets were frozen, resuspended in cold PBS (50 µl PBS per ml culture), then lysed by sonication on ice in 15 s bursts and the lysate clarified by centrifugation at 12 000×***g*** for 30 min at 4°C. The supernatant was supplemented with Glutathione Sepharose 4B resin (GE Healthcare; 1 ml 50% slurry per litre culture). The binding was allowed at 4°C on a rolling platform overnight. The beads were washed in phosphate-buffered saline (PBS) and the protein eluted in two successive 10 min incubation steps with 50 mM Tris–HCl, 10 mM reduced glutathione, pH 8.0. Proteolytic removal of the GST fusion protein partner was achieved by adding 200 µg PreScission protease (GE Healthcare) per 10 mg GST-rSUB1-ProM fusion protein. The protein mixture was transferred to a 7 kDa cut-off dialysis cassette and exchanged against 3 litres of 50 mM Tris–HCl, 150 mM NaCl, 1 mM EDTA, 1 mM DTT pH 7.0, for 2–4 h at 4°C, when the buffer was replaced and dialysis continued overnight. For each 10 ml of protein, 1 mini cOmplete protease inhibitor tablet (Roche) was added and the protein sample clarified by passage through a 0.22 µm filter. The protein was concentrated on a Centricon 70-plus ultrafiltration cartridge (10 kDa cut-off, Millipore) and finally polished by chromatography on a HiLoad Superdex 200 (26/60) prep grade size-exclusion column equilibrated in 20 mM HEPES, 150 mM NaCl, pH 7.4. The purified rSUB1-ProM eluted as a single peak which migrated as a doublet on reducing SDS–PAGE at the expected apparent molecular mass of ∼14 kDa. Protein gel slices were submitted for N-terminal sequencing (Alta Bioscience, U.K.) and five cycles of Edman degradation used to identify the N-terminal five amino acid residues of the smaller SUB1-ProM product.

### CD measurements

Far-UV circular dichroism (CD) spectra (200–260 nm) of purified rSUB1-ProM were recorded on a Jasco J-815 spectropolarimeter fitted with a cell holder, temperature controlled by a CDF-426S Peltier unit. Measurements were performed at 20°C at a protein concentration of 0.15 mg/ml in 20 mM HEPES pH 7.4, 35 mM NaCl, 2% glycerol, using fused silica cuvettes with 1 mm path length (Hellma, Jena, Germany). Spectra were recorded with 0.2 nm resolution and baseline corrected by subtraction of the appropriate buffer spectrum. CD intensities are presented as the mean residue CD extinction coefficient (Δε_mrw_) calculated as:

$$\Delta \varepsilon_{mrw}=\frac{S\cdot mrw}{32980\;\cdot c_{mg/ml}\cdot L}$$

where *S* is the signal in millidegrees, mrw is the mean residue weight (molecular mass divided by the number of residues), *c*_mg/ml_ is the concentration in mg/ml, and *L* is the pathlength (in cm). Secondary structure content was estimated using previously described methods [33].

### Protease inhibition assays and kinetic analysis

Assays of the proteolytic activity of rPfSUB1, subtilisin Carlsberg (SUBC, *bacillus licheniformis*; Sigma) and pronase (from *Streptomyces griseus*; Boehringer) were based on cleavage of the fluorogenic peptide substrate SERA4st1F-6R12 (Ac-CKITAQDDEESC-OH labelled on both cysteine sidechains with tetramethylrhodamine) [30]. The substrate exhibits low fluorescence in its intact state due to non-covalent, concentration-dependent dimerisation of the rhodamine moieties. Cleavage at any position within the peptide backbone allows dissociation of the rhodamine dimer, leading to a fluorescence increase [34]. Chymotrypsin-treated rPfSUB1 was stored as a 228 U/ml stock in 20 mM Tris–HCl pH 8.2, 150 mM NaCl, 10% glycerol. Purified rSUB1-ProM was stored as a stock solution (49.5 µM) in 20 mM HEPES, pH 7.4, 150 mM NaCl, from which it was diluted to final concentrations in the 7.81–250 nM range in the final reactions. For simple inhibition assays, PfSUB1 (0.045 units in 100 µl 25 mM Tris–HCl pH 8.2, 25 mM CHAPS, 12 mM CaCl_2_), or subtilisin Carlsberg (50 µg/ml in 100 µl 20 mM HEPES pH 7.4, 10 mM CaCl_2_) or pronase (0.1 mg/ml in 100 µl 20 mM Tris–HCl pH 8.2, 150 mM NaCl) in wells of white 96-well microtitre plates (Nunc) was pre-incubated with 0.4 µM rSUB1-ProM for 5 min then substrate (0.1 µM final) added and fluorescence increase continuously monitored with time at 21°C using a SpectraMax M5e plate reader with the SoftMax Pro 6.3 software as described previously [30], with readings taken every 3 to 5 min for 25–60 min using excitation and emission values of 552 nm and 580 nm, respectively. All experiments were performed in duplicate.

For determination of the apparent tight-binding inhibition constant, *K_i(app)_*, for inhibition of rPfSUB1 by rSUB1-ProM, the hydrolysis of SERA4st1F-6R12 by rPfSUB1 in the absence of SUB1-ProM was first monitored, followed by the addition of SUB1-ProM with negligible dilution of the reaction at a range of final concentrations (7.81 nM, 15.62 nM, 31.25 nM, 62.5 nM, 125 nM and 250 nM), thereafter allowing the rate of the reaction to reach a new steady state. The concentration of SERA4st1F-6R12 used was well below its *K*_m_, under which conditions *K_i(app)_* ∼ *K_i_* [35]. In the absence of inhibitor, the hydrolysis rate was linear over the selected time period and no more than 10% of the total available substrate was hydrolysed.

### SUB2-mediated MSP1 shedding assay

To evaluate the effects of rSUB1-ProM on PfSUB2-mediated shedding of the merozoite surface protein MSP1, highly mature synchronous *P. falciparum* 3D7 schizonts were washed and resuspended in protein-free RPMI 1640 medium with no additions, or EDTA (10 mM), or purified rSUB1-ProM (80 µg/ml), then incubated at 37°C for 30 min to allow egress to occur naturally. Residual schizonts were pelleted by centrifugation and clarified supernatant samples analysed by Western blot as described previously [10,22], probing with the mouse monoclonal antibody 89.1 (diluted 1 : 5000), which recognises an epitope within the N-terminal segment of MSP1, or the human monoclonal antibody X509 (1 : 1000), which recognises an epitope close to the C-terminal region of MSP1.

### Generation of transgenic *P. falciparum*

A PF3D7_0507400 seed (nucleotides 298–317) was cloned into a dual Cas9-seed plasmid (pDC2-cam-Cas9-U6-hDHFR; kindly provided by Marcus Lee, Sanger Institute, U.K.) using InFusion cloning (Takara Clontech) with complementary seed primers (5′-AAGTATATAATATTGTCAAAACTTATGAATTCTTGGTTTTAGAGCTAGAA-3′ and 5′-TTCTAGCTCTAAAACCAAGAATTCATAAGTTTTGACAATATTATATACTT-3′). For the donor repair plasmid, PF3D7_0507400 nucleotides 52 to 320 were used as the 5′ homology region. Silent mutations were introduced at nucleotides 303, 306, 312 and 315 (within the seed sequence). Sequence encompassing nucleotides 321 to the end of the ORF were re-codonised. The *P. falciparum SERA2* intron containing an internal *loxP* site (*loxPint*) [36] was inserted after base 381. A stop codon, a second *loxP* site and 3′ homology region (900 bp of *P. falciparum* sequence beyond the PF3D7_0507400 stop codon) were placed immediately following the re-codonised sequence. The truncated SUB1-ProM protein product predicted to be expressed from the excised locus would end at residue 127 and be terminated after a further eight residues coded by intron sequence. For introduction of the plasmids into *P. falciparum*, 10 µg donor plasmid was linearised using PstI (Thermo Fisher Scientific) and transfected by electroporation along with 10 µg circular Cas9 seed plasmid into the *P. falciparum* 3D7 DiCre-expressing B11 clone, as described previously [31]. Cloned lines from two independent transfection experiments were generated by limiting dilution. Correct splicing of the *loxPint* intron in the parasite clones was confirmed by nucleotide sequencing of gel-purified RT-PCR product.

### Growth rate quantification of transgenic *P. falciparum*

Ring-stage parasites of four transgenic clones from two independent transfections (as well as a fifth clone that had not sustained modification of the PF3D7_0507400 locus and so acted as a wild-type control for PCR purposes) were treated for 4 h by incubation in medium containing 100 nM rapamycin in 0.1% v/v DMSO, or DMSO only (vehicle only control) for 4 h, after which time the drug (or solvent) was washed out. At 24 h, cultures were sampled for diagnostic PCR to confirm modifications to the locus. At 48 h, parasitaemia values were calculated and normalised to 0.1% and/or 1%. After a further 48 h (0.1% and 1% starting parasitaemia cultures) and again at 96 h (0.1% starting parasitaemia cultures), cultures were sampled, stained with Vybrant Green (Thermo Fisher Scientific), and DNA-positive events quantified by flow cytometry (BD Aria Fusion).

## Results

### Identification of a conserved *Plasmodium* gene encoding a subtilisin propeptide-like protein

**Figure 1.**
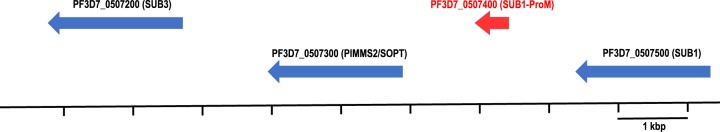
The *P. falciparum* PF3D7_0507400 gene is flanked by three genes encoding subtilisin-like proteins and is located in a region of high synteny across the *Plasmodium* genus. Architecture of *P. falciparum* 3D7 chromosome 5 in the vicinity of the PF3D7_0507400 gene. The *SUB1*, *PIMMS2*/*SOPT* and *SUB3* genes (blue) flank the PF3D7_0507400 locus (red). Arrows denote the direction of transcription. The entire locus is highly syntenic across all sequenced *Plasmodium* genomes (see PlasmoDB; http://plasmodb.org/plasmo/).

*P. falciparum* gene PF3D7_0507400 was initially highlighted as part of a genome-wide bioinformatic screen for blood-stage parasite genes that have a transcriptional profile similar to that of the invasion-associated merozoite protein apical membrane antigen-1 (AMA1) [37], and that contain a predicted signal sequence or transmembrane domain. PF3D7_0507400 comprises a single exon on chromosome 5 lying between the *SUB1* gene (PF3D7_0507500) and the *PIMMS2*/*SOPT* gene (PF3D7_0507300) (Figure 1). This is followed in turn by the *SUB3* gene (PF3D7_0507200), with all four genes being transcribed in the same direction. Annotated in PlasmoDB (http://plasmodb.org/plasmo/) as a conserved gene of unknown function, PF3D7_0507400 has clear orthologues at highly syntenic locations in all *Plasmodium* genomes examined.

**Figure 2.**
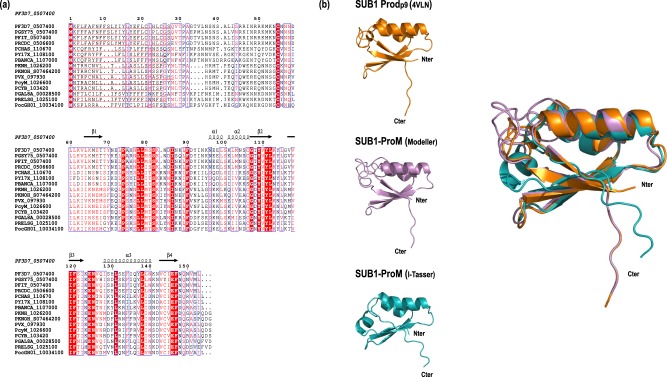
The PF3D7_0507400 gene encodes a conserved subtilisin propeptide-like protein. (**a**) Multiple alignment (performed using ESPript 3.0; http://espript.ibcp.fr) [63] of the predicted primary amino acid sequences of PF3D7_0507400 (SUB1-ProM) and several *Plasmodium* orthologues. N-terminal secretory signal sequences predicted by SignalP 4.1 [64] are underlined. Blue boxes indicate positions of >70% similarity (also shown in red text) or identity (white text on red shading) (see also Supplementary Figure S1). Predicted secondary structure elements are indicated. Note that whilst the current PlasmoDB annotations suggest that some of the PF3D7_0507400 orthologues contain introns, our analysis suggests that this is incorrect, and the predicted translation products used for this analysis are based on each gene comprising just a single exon. *Plasmodium* species and strains acronyms are: PF3D7, *P. falciparum* (3D7); PGSY75, *P. gaboni*; PFIT, *P. falciparum* (IT); PRCDC, *P. reichenowi* (CDC); PCHAS, *P. chabaudi chabaudi*; PY17X, *P. yoelii* (17X); PBANCA, *P. berghei* (ANKA); PKNH, *P. knowlesi* (H); PKNOH, *P. knowlesi* (Malayan Strain Pk1 A); PVX, *P. vivax* (Sal-1); PcyM, *P. cynomolgi* (M); PCYB, *P. cynomolgi* (B); PGAL8A, *P. gallinaceum* (8A); PRELSG, *P. relictum* (SGS1-like); PocGH01, *P. ovale curtisi* (GH01). (**b**) Cartoon representation of the PfSUB1 propeptide core Prod_p9_ (orange), shown in isolation from the PfSUB1-Fab complex X-ray crystal structure (PDB ID: 4LVN). Below, two models of the PF3D7_0507400 gene product (called SUB1-ProM; see main text), also depicted as cartoons. The first model (violet; Val63–Leu153) was generated with pGenTHREADER/Modeller, whilst the second (Teal blue; Asn77–Leu153) was from I-Tasser. Right, superimposition of the three cartoons to show spatial agreement. N- and C-termini of each modelled protein are indicated. Superimposition of the Modeller and I-Tasser models with 4LVN showed a deviation of 0.49 Å and 1.07 Å, respectively (RMSD value on Cα).

PF3D7_0507400 encodes a 153-residue protein with a predicted 27-residue secretory signal peptide (SignalP 4.1; [38]) and a total predicted molecular mass of ∼17.9 kDa. Alignment of the predicted protein products of several of these orthologues (Figure 2a and Supplementary Figure S1) reveals conservation of several primary structural features, including the signal peptide. The orthologues display no predicted transmembrane domain or predicted glycosyl phosphatidylinositol anchor signal and share particularly high identity within the C-terminal region of each protein. BLAST analysis of the non-redundant protein databases with the predicted PF3D7_0507400 primary sequence revealed no significant similarities beyond the *Plasmodium* orthologues. However, submission of the predicted mature *P. falciparum* protein sequence (Gln28–Leu153) to protein fold prediction using pGenTHREADER [39] in combination with Modeller v9.20 (https://salilab.org/modeller/) [40] identified in each case the X-ray crystal structures of PfSUB1 (PDB ID: 4LVN) [14] and *P. vivax* SUB1 (PvSUB1; PDB ID: 4TR2) [41] as the best templates. Examination using the pGenTHREADER/Modeller programmes of even the most divergent orthologue relative to the *P. falciparum* 3D7 gene (*P. yoelii* 17X PY17X_1108100; 28.67% identity; Supplementary Figure S1), again identified the PfSUB1 structure as the best structural template. Predicted structural homology was restricted to the propeptides of both *Plasmodium* subtilisins (called Prod_p9_ in the case of PfSUB1; [14]). Similar to previously determined family S8 bacterial and mammalian subtilisin propeptide structures (e.g. [42–44]) as well as the *P. falciparum* SUB2 (PfSUB2) propeptide [45] and the stand-alone propeptide mimic TgMIC5 (PDB ID: 2LU2) [7], the core of both SUB1 propeptides comprises two antiparallel alpha helical elements, one of which is slightly kinked, packed against a four-stranded antiparallel beta sheet to produce an overall compact βαβ–βαβ topology (Figure 2b and Supplementary Figure S2).

To further investigate potential structural features of the PF3D7_0507400 and PY17X_1108100 gene products, the sequences were submitted to analysis using I-Tasser (Iterative Threading ASSEmbly Refinement; https://zhanglab.ccmb.med.umich.edu/I-TASSER/) which uses a meta-threading method for template-based protein structure prediction. The resulting PF3D7_0507400 I-Tasser model showed a confidence score of −3.49 (C-scores ranging from −5 to 2 for best quality models) with an estimated TM-score of 0.33 ± 0.11. Co-ordinates of the model were fed into the PDBeFold server (http://www.ebi.ac.uk/msd-srv/ssm/) to search for a similar fold in the PDB, identifying the PfSUB1 propeptide and *T. gondii* MIC5 amongst the top three solutions. Superimposition of the Modeller and I-Tasser PF3D7_0507400 models with the PfSUB1 propeptide structure resulted in RMSD values of 0.4 Å and 1.07 Å, respectively (Figure 2b). The same strategies were used to model the gene product of PY17X_1108100, once again identifying the PfSUB1 prodomain as the best template (Supplementary Figure S2).

**Figure 3.**
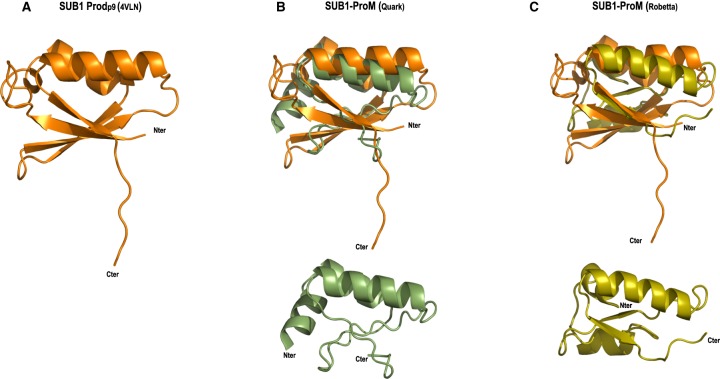
*Ab initio* structure prediction of PF3D7_0507400 (SUB1-ProM) indicates structural similarity to the PfSUB1 propeptide. (**a**) Cartoon representation of the PfSUB1 propeptide core Prod_p9_ (orange), shown in isolation from the PfSUB1-Fab complex X-ray crystal structure (PDB 4LVN). (**b**) Top, cartoon representation of (**a**) superimposed onto the *ab initio* predicted model of SUB1-ProM (Asn87–Leu153) from QUARK (olive green). Below, the QUARK *ab initio* model is shown in isolation. (**c**) Cartoon representation of (**a**) superimposed onto the *ab initio* predicted model of SUB1-ProM (Asn87–Leu153) from Robetta (yellow). Below, the Rosetta *ab initio* model is shown in isolation. N- and C-termini of each modelled protein are indicated.

As a final, non-template-based approach to investigating the predicted structure of PF3D7_0507400, the sequence was analysed using two *ab initio* modelling servers. Submission to the Robetta protein structure prediction server (http://robetta.bakerlab.org), which uses the Rosetta software package for *de novo* prediction, generated a model with a confidence score of 0.34 (1 being the best possible score) corresponding to the average pairwise TM-score (a measure of global fold similarity) of the top 10 Rosetta models. The QUARK server (https://zhanglab.ccmb.med.umich.edu/QUARK/) was similarly used as an additional *ab initio* protein structure prediction method due to its excellent performance in the CASP9 and CASP10 (Critical Assessment of Structure Prediction) experiments (Protein Structure Prediction Center; http://predictioncenter.org/). The co-ordinates of the QUARK model were fed into the PDBeFold server (www.ebi.ac.uk/msd-srv/ssm/) to search for a similar fold in the PDB; the PfSUB1 propeptide structure (4LVN) was present amongst the solutions. The C-terminal region of the Robetta and QUARK models (Asn87–Leu153) superimposed onto the PfSUB1 propeptide structure with RMSD values of 7.5 Å and 3.25 Å, respectively (Figure 3).

Collectively, these approaches suggested structural similarity between PF3D7_0507400 (and its orthologues) and subtilase propeptides, with a particularly close structural relationship to the SUB1 propeptide. Because of this similarity, further supported by evidence presented below, the PF3D7_0507400 gene product is henceforth referred to as SUB1-ProM (SUB1 propeptide mimic).

### SUB1-ProM is expressed in asexual blood stages of *P. falciparum*

The transcriptional profile of the *P. falciparum* SUB1-ProM gene obtained from previous global transcriptome studies e.g. [46,47] (data available in PlasmoDB) indicated that PF3D7_0507400 is transcribed in asexual blood stages, with peak expression in mature schizonts. To evaluate expression at the protein level we examined a recently obtained global proteomic dataset [48]. This clearly identified with high confidence two tryptic peptides of SUB1-ProM (corresponding to 17.6% coverage) in extracts of *P. falciparum* asexual blood stage schizonts (Supplementary Figure S3), confirming that the protein is expressed in these clinically relevant developmental stages of the parasite lifecycle.

**Figure 4.**
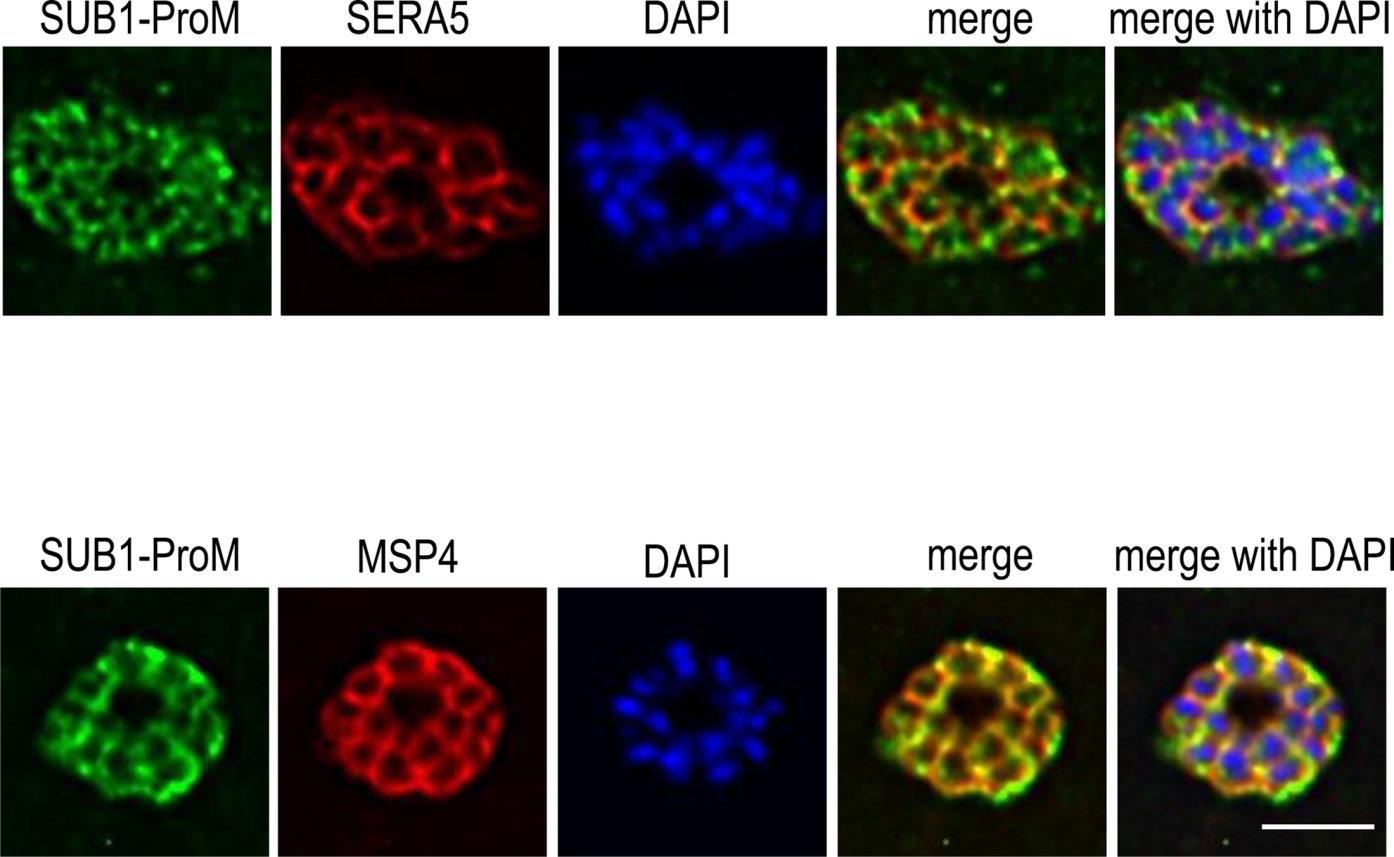
SUB1-ProM is expressed in the PV or at the merozoite surface in *P. falciparum* schizonts. IFA of mature *P. falciparum* 3D7 schizonts probed with the indicated antibodies. The merged images are presented both with and without the nuclear stain DAPI. The anti-SERA5 pattern is typical of a PV location, whilst the merozoite surface protein MSP4 delineates the plasma membrane of intracellular segmented merozoites. Note that the distance between the merozoite plasma membrane and PV membrane is generally too small to be resolved by light microscopy. Both signals show extensive co-localisation with the SUB1-ProM signal, which is more evident with the anti-MSP4 signal. Scale bar, 5 µm.

The presence of a predicted secretory signal peptide in the SUB1-ProM sequence (and the absence of apparent membrane-binding structures) is consistent with the protein being directed to the PV, since this is the default location for soluble secreted proteins in the malaria parasite [49]. To experimentally investigate the expression and subcellular localisation of SUB1-ProM, we expressed recombinant SUB1-ProM in *Escherichia coli* as a glutathione S-transferase (GST) fusion protein (called GST-rSUB1-ProM) and raised polyclonal antibodies to the purified protein. Analysis of parasite extracts by Western blot with these antibodies failed to detect a specific signal. However, use of the antibodies to probe fixed and permeabilized *P. falciparum* schizonts in indirect immunofluorescence assays (IFA) (Figure 4 and Supplementary Figure S4) produced a signal typical of a PV or merozoite surface location, overlapping with the established PV marker SERA5 (which is also a major endogenous substrate of PfSUB1 [19]) and with the merozoite surface protein MSP4. Distinguishing between a merozoite surface and PV localisation by light microscopy in *Plasmodium* blood stages is challenging, but given the predicted soluble nature of SUB1-ProM it was concluded that SUB1-ProM is likely expressed in the PV or at the merozoite surface periphery.

### Key structural features of SUB1 Prod_p9_ are conserved in SUB1-ProM

**Figure 5.**
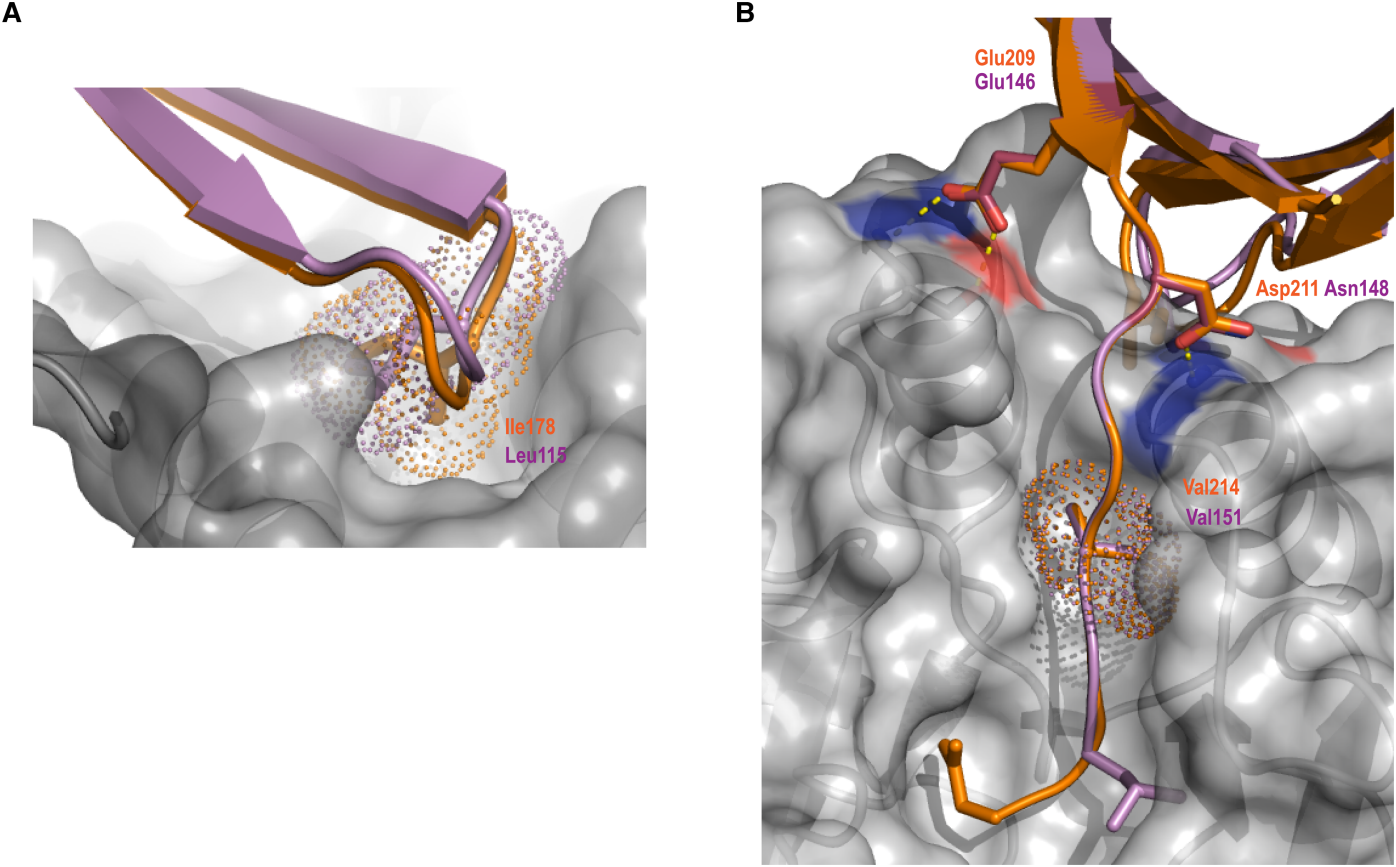
SUB1-ProM shares conserved features of the PfSUB1 propeptide required for interaction with the PfSUB1 catalytic domain. (**a**) Superimposition of the pGenTHREADER/Modeller SUB1-ProM model (violet cartoon) onto the X-ray crystal structure of PfSUB1 Prod_p9_ (orange) complexed to the PfSUB1 catalytic domain (grey molecular surface) (PDB ID: 4LVN). This view faces the back of the active site in order to illustrate a structurally conserved hydrophobic plug involving Prod_p9_ Ile178/SUB1-ProM Leu115 which inserts into a hydrophobic pocket in the PfSUB1 catalytic domain. (**b**) Rotation of the previous view to face the active site groove, illustrating conservation of the helix capping residues Glu209/Asp211 in Prod_p9_ and Glu146/Asn148 in SUB1-ProM. Hydrogen bond interactions with the PfSUB1 catalytic core are indicated (yellow dotted lines). The extreme C-terminal residues of Prod_p9_ (Asp217) and SUB1-ProM (Leu153) are shown extending into the active site. The side chains of the positionally conserved P4 Val (Prod_p9_ Val214 and SUB1-ProM Val151) are shown as sticks within their dotted surface area, filling the S4 pocket of the PfSUB1 active site cleft. Note that the SUB1-ProM C-terminal tail is one residue shorter than the Prod_p9_ tail and does not fulfil the canonical PfSUB1 active site S2 pocket requirements (Gly or Ala only), indicating a possible non-canonical active site interaction with the PfSUB1 catalytic domain. The SUB1-ProM capping residues (Glu 146, Asn 148) and the SUB1-ProM hydrophobic plug (Leu118) were also structurally conserved as compared with the PfSUB1 propeptide structure 4LVN in both the Robetta and QUARK models of SUB1-ProM.

In the light of the structure-based comparisons identifying the PfSUB1 prodomain Prod_p9_ as the best template for SUB1-ProM, we further examined the predicted structural similarities between these proteins. Like other subtilisins, immediately following cleavage at the propeptide-catalytic domain junction, the propeptides of PfSUB1 and PvSUB1 remain tightly bound to their cognate catalytic domain, primarily through interactions between the beta sheet of the propeptide and two prominent surface-located parallel alpha helices of the catalytic domain [14,41]. In the case of PfSUB1, key contributions to propeptide binding include hydrophobic interactions with the sidechain of Ile178, located at the beta hairpin turn linking two strands of the Prod_p9_ beta sheet, which plugs into a hydrophobic pocket on the catalytic domain surface (Figure 5a and Supplementary Figure S5a). Interestingly, Prod_p9_ Ile178 is replaced by a Leu residue (Leu115) in SUB1-ProM, the sidechain of which could be accommodated in the PfSUB1 catalytic domain hydrophobic pocket in a very similar manner; this residue is also highly conserved in the *Plasmodium* SUB1-ProM orthologues, being replaced by a Val or Ile in only two cases (Figure 2a). On the opposite side of the Prod_p9_-SUB1 catalytic domain interface, the sidechains of Prod_p9_ residues Glu209 and Asp211 engage in H-bond interactions with main chain and sidechain atoms at the ends of the long helices on the catalytic domain, capping these helices (Figure 5b and Supplementary Figure S5b). Similar helix-capping interactions have been observed in other subtilisin-propeptide complexes, including that of bacterial subtilisin BPN’ where propeptide residues Glu69 and Asp71 perform this capping function [43,50]. The equivalent residues in SUB1-ProM, Glu146 and Asn148, could potentially engage in similar helix-capping H-bond interactions, in agreement with the predicted propeptide-like structure of SUB1-ProM (Figure 5b); notably, Glu146 is completely conserved in all *Plasmodium* SUB1-ProM orthologues, whilst Asn148 is replaced by an Asp in some orthologues (Figure 2a). Known subtilisin propeptide-catalytic domain interactions are further reinforced by extension of the C-terminal tail of the propeptide into the active site groove of the catalytic domain, interacting with the non-prime side pockets in an extended, substrate-like manner e.g. [14,41,43,51]. In the case of PfSUB1, the C-terminal tail of the propeptide enhances its inhibitory affinity but importantly is not essential for inhibition [52]. Our modelling exercise suggests that SUB1-ProM possesses a similar C-terminal extension (Figure 5b). This does not completely mimic the Prod_p9_ tail, which completely fills the non-prime side (S4–S1) substrate-binding pockets due to it being derived from autocatalytic cleavage of the precursor zymogen [14]. However, the SUB1-ProM C-terminus shares features with the Prod_p9_ tail, including the presence of a Val residue (Val151) which can be modelled into the important and relatively deep S4 binding pocket of the PfSUB1 catalytic domain [14].

It was concluded that SUB1-ProM constitutes a malarial subtilisin propeptide-like protein that is highly conserved across the genus and that has particularly close structural relatedness to the SUB1 propeptide.

### SUB1-ProM is a rapid, potent and selective inhibitor of PfSUB1 peptidase activity

As mentioned in the Introduction, a recently characterised example of a stand-alone subtilisin propeptide-like protein is TgMIC5 of the parasitic protist *T. gondii*, an organism closely related to *Plasmodium*. TgMIC5 regulates the activity of a *T. gondii* subtilase called TgSUB1, that cleaves parasite surface proteins involved in motility and host cell invasion [8]. TgSUB1 is not strictly a functional homologue of *Plasmodium* SUB1, since TgSUB1 has no known role in egress of *T. gondii* from host cells. However, these observations along with our localisation of SUB1-ProM to the PV where it could potentially interact with discharged PfSUB1, prompted us to examine the possibility that SUB1-ProM might act similarly to TgMIC5 and inhibit the activity of endogenous PfSUB1.

**Figure 6.**
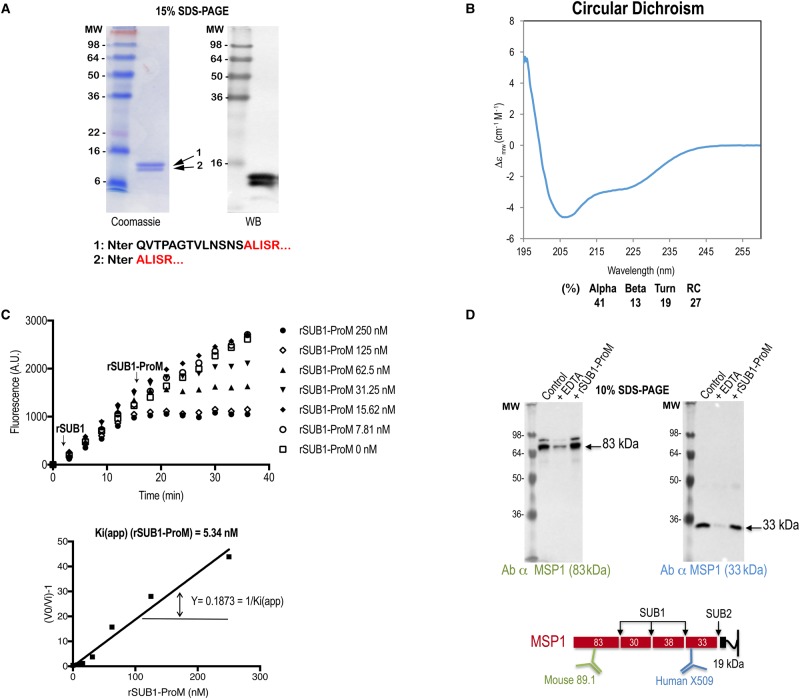
SUB1-ProM is a potent, fast-binding inhibitor of PfSUB1 but not of PfSUB2. (**a**) Left: Coomassie-stained SDS–PAGE analysis of purified *P. falciparum* rSUB1-ProM. Indicated below are the N-terminal sequences of the two components of the doublet (arrowed) as determined by Edman degradation. The faster migrating species lacked the N-terminal 13 residues of the higher molecular mass form, indicating limited N-terminal truncation during purification. Right: Western blot, showing reactivity of a pool of mouse antisera raised against purified GST-rSUB1-ProM. (**b**) Far-UV CD spectrum of rSUB1-ProM in solution at room temperature, indicating that the protein is folded with an alpha/beta secondary structure consistent with that predicted by modelling (e.g. the I-Tasser full-length model predicts 41% alpha, 13% beta and 46% turn plus random coil). (**c**) Inhibition of rPfSUB1 activity by rSUB1-ProM. A solution of fluorogenic substrate SERA4st1F-6R12 (0.1 µM) in 25 mM Tris–HCl pH 8.2, 25 mM CHAPS, 12 mM CaCl_2_ was supplemented with rPfSUB1 (0.045 U in 100 µl) and the resulting fluorescence increase monitored over the ensuing 15–18 min. At this time, 1 µl of serially diluted rSUB1-ProM was added with rapid mixing (arrowed) and the hydrolysis rate measured for a further 20 min. Reaction velocities prior to and following rSUB1-ProM addition were measured and the *K_i(app)_* calculated as indicated (in the typical experiment shown here the calculated value was 5.34 nM). The experiment was repeated five times, resulting in a mean experimentally determined *K_i(app)_* of 5.67 ± 0.25 nM. (**d**) rSUB1-ProM has no effect on *P. falciparum* SUB2 activity. Western blot analysis of SUB2-mediated shedding of MSP1 from naturally released *P. falciparum* merozoites. Merozoite release was allowed to take place in the presence of 10 µM EDTA (positive control inhibitor) or 4.95 µM rSUB1-ProM. Culture supernatants containing shed MSP1 fragments were then analysed by Western blot, probing with two different monoclonal antibodies (89.1 or X509) that recognise either an 83 kDa shed fragment or a 33 kDa shed fragment. Whilst EDTA strongly inhibited shedding as expected, rSUB1-ProM had no discernible effect on MSP1 shedding.

To do this, we took advantage of the *E. coli*-derived GST-rSUB1-ProM. Cleavage of this protein at an internally engineered cleavage site allowed the production of highly purified rSUB1-ProM lacking the GST fusion partner and the predicted signal peptide. This protein, which was fully soluble, migrated on SDS–PAGE as a closely spaced doublet, indicating heterogeneity (Figure 6a). N-terminal sequencing by Edman degradation showed that this was due to limited N-terminal truncation of a fraction of the protein during purification. The polyclonal antibodies generated against the GST-rSUB1-ProM fusion protein recognised both species as expected (Figure 6a). Examination of the recombinant protein by CD (Figure 6b) revealed a secondary structure content fully in accord with the predicted subtilisin propeptide-like fold. However, exhaustive attempts to confirm this by X-ray crystallography failed to obtain diffracting crystals of either GST-rSUB1-ProM or the cleaved, GST-free form of the protein.

To examine the capacity of SUB1-ProM to inhibit PfSUB1 enzyme activity, we used a previously described kinetic approach [52] in which varying amounts of rSUB1-ProM were added to ongoing hydrolysis reactions containing recombinant PfSUB1 (rPfSUB1) in the presence of a well-characterised fluorogenic peptide substrate based on a cleavage site within the endogenous protein substrate SERA4 [19]. This method enables the calculation of apparent *K_i_* values (*K_i(app)_*) based on the change in reaction velocity upon test inhibitor addition. As shown in Figure 6c, the addition of rSUB1-ProM resulted in rapid, concentration-dependent decreases in hydrolysis rate, with no evidence of a hyperbolic transition period, indicating that rSUB1-ProM is a rapid, competitive inhibitor of PfSUB1. Based on the kinetic data we calculated a *K_i(app)_* of 5.67 ± 0.25 nM, which is very close to the previously determined value of 5.3 ± 0.4 nM for the PfSUB1 propeptide [52]. These results confirmed rSUB1-ProM as a potent, fast-binding inhibitor of PfSUB1.

Aside from PfSUB1, the only other subtilase that has been shown to play an important role in the asexual blood stage lifecycle of *P. falciparum* is PfSUB2, which sheds merozoite surface proteins during RBC invasion in a calcium-dependent manner [10]. PfSUB2 has not been expressed in an enzymatically active recombinant form, but its activity can readily be measured *in vitro* by the shedding of its native parasite protein substrates from the surface of free merozoites [10,53]. These substrates include merozoite surface protein-1 (MSP1), an abundant glycolipid-anchored merozoite surface protein. To examine the capacity of SUB1-ProM to modulate PfSUB2 activity, we used a simple cell-based assay in which merozoite egress and subsequent MSP1 shedding was allowed to take place directly into medium containing rSUB1-ProM. As shown in Figure 6d, even high concentrations of rSUB1-ProM had no discernible effect on the efficiency of MSP1 shedding, whereas the calcium chelating agent EDTA displayed the expected potent inhibition. These results suggested that SUB1-ProM does not inhibit PfSUB2 activity. It is worth noting that we have previously shown that whilst the PfSUB2 propeptide efficiently inhibits PfSUB2-mediated MSP1 shedding, it does not inhibit PfSUB1 [10].

To further assess the selectivity of rSUB1-ProM, we examined its capacity to inhibit the activity of subtilisin Carlsberg from *Bacillus licheniformis*, as well as pronase, which consists of a mixture of bacterial endo- and exo-peptidases. No inhibition of any of these proteases was detected even at the highest concentrations tested (Supplementary Figure S6). Collectively, these data suggested that SUB1-ProM is a highly selective inhibitor of SUB1.

### The SUB1-ProM capping residues and C-terminal segment are not required for parasite growth

Recent genome-wide screens of gene essentiality in *Plasmodium spp.* have indicated that SUB1-ProM is dispensable for parasite proliferation *in vitro* [54,55]. However, given the ubiquitous requirement for tight regulation of the proteolytic activity of subtilisins, we sought to assess the influence of the SUB1-ProM capping and C-terminal residues on parasite growth through a direct comparison of transgenic parasite lines expressing wild-type or C-terminally truncated forms of SUB1-ProM. Conditional genetic recombination was used truncate SUB1-ProM in *in vitro* cultured *P. falciparum* parasites, such that they lacked sequence encoding the SUB1-ProM C-terminus (Supplementary Figure S7a), including the capping residues and Val151. In an analysis of multiple independent clones (Supplementary Figure S7b), we found that conditional truncation of the C-terminus of SUB1-ProM had no effect on parasite growth rates (Supplementary Figure S7c).

## Discussion

It has been widely noted as remarkable that, despite their conserved core fold, subtilisin propeptides generally share little primary sequence homology. The PfSUB1 propeptide and SUB1-ProM described here provide excellent examples of that. Notwithstanding their low sequence similarity, our comparison of the modelled SUB1-ProM structure and its other *Plasmodium* orthologues with the known structure of Prod_p9_ revealed conservation of specific features known to be critical for the interaction between the PfSUB1 propeptide and its cognate catalytic domain. The structural modelling was strongly supported by our experimental results showing unambiguously that SUB1-ProM is a potent and selective inhibitor of PfSUB1 enzyme activity, whilst our mass spectrometric and localisation data implicated SUB1-ProM as a PV protein in asexual blood stage schizonts.

The gene encoding SUB1-ProM lies within, and is in the same orientation as, an array of three of the four *Plasmodium* SUB genes, in a configuration reminiscent of an operon structure. The vast majority of eukaryotic genomes lack the operon architecture of prokaryotes, in which functionally related genes are clustered and co-transcribed. Nonetheless, some studies have detected a tendency for functionally related eukaryotic genes to cluster on chromosomes, perhaps in order to fulfil a requirement for transcriptional co-regulation e.g. [56]. With this in mind, the chromosomal proximity of PF3D7_0507400 to the adjacent three subtilisin (or subtilisin-like) *P. falciparum* genes, and the high degree of synteny across *Plasmodium* genomes at this locus, is intriguing. It is tempting to speculate that the close linkage between the *SUB1* and *SUB1-ProM* genes is indicative of a need for closely synchronised expression of both genes, consistent with a functional association.

It is interesting to speculate on the physiological role SUB1-ProM might play in the parasite. The malaria parasite replicates by an unusual process termed schizogony, in which repeated rounds of nuclear division generates a multinucleated schizont bounded by a single plasma membrane. The various intracellular organelles are then segregated along with invagination of the plasma membrane (a process often referred to as segmentation) to produce individual mature merozoites. Premature triggering of egress could therefore result in the release of improperly segmented - and thus non-invasive - merozoites. Indeed, experimental treatment of mature schizonts with phosphodiesterase inhibitors, which artificially induce PfSUB1 discharge by up-regulation of parasite cGMP levels, results in the massive premature release of largely non-invasive merozoites [57,58]. There is therefore a clear biological necessity for the temporally very tightly regulated nature of egress. It could be envisaged that mechanisms to suppress spurious, untimely low-level SUB1 activity in the PV (e.g. due to ‘leaky’ premature exoneme discharge) might be important to prevent potentially catastrophic premature activation of the egress pathway. We propose that SUB1-ProM may play such a regulatory role in the PV or at the intracellular merozoite surface (which is effectively exposed to the contents of the PV lumen). This would be by no means unprecedented; endogenous protease inhibitors are widespread in biological systems, where they often play crucial roles, and although protein-based peptidase inhibitors are relatively sparse in prokaryotes and unicellular eukaryotes [59], they have previously been identified in the malaria parasite for other peptidase classes [60–62]. Our finding that the PfSUB1 and PvSUB1 propeptides were the best templates for homology modelling of SUB1-ProM may indicate an evolutionary adaptation of SUB1-ProM to optimally mimic these propeptides in order to perform its regulatory function. In apparent conflict with this notion is the structure of the C-terminal tail of SUB1-ProM, which contains conserved residues involved in interactions with SUB1, but which varies in length in different orthologues and which does not appear to completely fill the active site groove when modelled in complex with PfSUB1. Our own previous studies have shown that the C-terminal tail of the PfSUB1 propeptide is not essential for inhibition [52], and comparative analysis of several *Plasmodium* SUB1 orthologues have indicated subtle differences in their substrate specificity [30]. Collectively, this may explain the observed variation in the C-terminal tail sequences of the SUB1-ProM orthologues, each of which may be optimally adapted to its cognate SUB1 orthologue. It may be critical that SUB1-ProM is not too potent an inhibitor, since its proper functioning may require a fine balance between efficient inhibition of low-level, premature SUB1 activity in the PV yet without blocking eventual egress.

We observed no effect of truncation of the SUB1-ProM C-terminus on parasite growth *in vitro*. Combined with existing data from global evaluations of gene essentiality in *Plasmodium* [54,55], these results suggest that any contribution of SUB1-ProM to overall parasite fitness may be relatively subtle, or that any essential functions of SUB1-ProM do not require the C-terminal region of the protein. Given that SUB1 is responsible for the maturation of a range of merozoite antigens prior to exposure of the merozoite to the host immune system, it will be important to establish the importance of SUB1 regulation by SUB1-ProM in the presence of physiological selective pressures.

In conclusion, we have identified a putative endogenous inhibitor of the essential egress-related protease SUB1, expanding our understanding of the mechanisms underlying subtilase function in this important intracellular pathogen.

**Key points**

- Malaria parasites use a subtilisin-like protease, SUB1, to egress from red cells
- We identify a stand-alone malarial subtilisin propeptide-like protein, SUB1-ProM
- SUB1-ProM is likely expressed in the lumen of the parasitophorous vacuole
- SUB1-ProM is a potent, selective inhibitor of SUB1
- SUB1-ProM may be an endogenous regulator of SUB1 that prevents premature egress

## Abbreviations

AMA1: apical membrane antigen-1
CD: circular dichroism
DAPI: 4,6-diamidino-2-phenylindol
IFA: immunofluorescence analysis
MSP: merozoite surface protein
PBS: phosphate-buffered saline
PV: parasitophorous vacuole
RBC: red blood cells
