# Supplementary material for: A malaria parasite subtilisin propeptide-like protein is a potent inhibitor of the egress protease SUB1
Source: Biochem J. 2020 Jan 31;477(2):525–40. doi: 10.1042/BCJ20190918 (PMC6993865; doi:10.1042/BCJ20190918)
Supplement: Supplementary Figures S1-S7 [file BCJ-477-525-s1.pdf]

# Supplemental data

**Percent Identity Matrix (Clustal2.1):**

| | | | | | | | | | | | | | | | | | |
|---|---|---|---|---|---|---|---|---|---|---|---|---|---|---|---|---|---|
| 1: PF3D7_0507400 | 100.00 | 90.20 | 100.00 | 96.73 | 30.67 | 30.30 | 28.67 | 29.33 | 34.48 | 34.48 | 37.93 | 38.62 | 38.62 | 38.41 | 39.74 | 42.42 | 40.00 |
| 2: PGSY75_0507400 | 90.20 | 100.00 | 90.20 | 87.58 | 31.33 | 31.06 | 29.33 | 30.00 | 33.10 | 33.10 | 36.55 | 37.24 | 37.24 | 37.75 | 39.07 | 43.94 | 40.67 |
| 3: PFIT_0507400 | 100.00 | 90.20 | 100.00 | 96.73 | 30.67 | 30.30 | 28.67 | 29.33 | 34.48 | 34.48 | 37.93 | 38.62 | 38.62 | 38.41 | 39.74 | 42.42 | 40.00 |
| 4: PRCDC_0506600 | 96.73 | 87.58 | 96.73 | 100.00 | 31.33 | 31.06 | 29.33 | 30.00 | 33.10 | 33.10 | 36.55 | 37.24 | 37.24 | 37.75 | 39.07 | 41.67 | 39.33 |
| 5: PCHAS_110670 | 30.67 | 31.33 | 30.67 | 31.33 | 100.00 | 91.79 | 84.21 | 82.89 | 37.67 | 37.67 | 36.99 | 39.73 | 39.73 | 39.47 | 39.47 | 44.78 | 47.02 |
| 6: YYG_01218 | 30.30 | 31.06 | 30.30 | 31.06 | 91.79 | 100.00 | 87.31 | 85.82 | 38.46 | 38.46 | 38.46 | 41.54 | 41.54 | 42.54 | 42.54 | 42.54 | 47.76 |
| 7: PY17X_1108100 | 28.67 | 29.33 | 28.67 | 29.33 | 84.21 | 87.31 | 100.00 | 91.45 | 35.62 | 35.62 | 34.93 | 37.67 | 37.67 | 37.50 | 37.50 | 42.54 | 41.72 |
| 8: PBANCA_1107000 | 29.33 | 30.00 | 29.33 | 30.00 | 82.89 | 85.82 | 91.45 | 100.00 | 36.99 | 36.99 | 35.62 | 38.36 | 38.36 | 36.84 | 38.16 | 41.79 | 41.06 |
| 9: PKNH_1026200 | 34.48 | 33.10 | 34.48 | 33.10 | 37.67 | 38.46 | 35.62 | 36.99 | 100.00 | 100.00 | 79.19 | 82.55 | 82.55 | 34.23 | 40.94 | 50.76 | 44.52 |
| 10: PKNOH_S07464200 | 34.48 | 33.10 | 34.48 | 33.10 | 37.67 | 38.46 | 35.62 | 36.99 | 100.00 | 100.00 | 79.19 | 82.55 | 82.55 | 34.23 | 40.94 | 50.76 | 44.52 |
| 11: PVX_097930 | 37.93 | 36.55 | 37.93 | 36.55 | 36.99 | 38.46 | 34.93 | 35.62 | 79.19 | 79.19 | 100.00 | 85.23 | 85.23 | 35.57 | 40.27 | 51.52 | 44.52 |
| 12: PcyM_1026600 | 38.62 | 37.24 | 38.62 | 37.24 | 39.73 | 41.54 | 37.67 | 38.36 | 82.55 | 82.55 | 85.23 | 100.00 | 100.00 | 38.93 | 44.97 | 53.03 | 45.89 |
| 13: PCYB_103420 | 38.62 | 37.24 | 38.62 | 37.24 | 39.73 | 41.54 | 37.67 | 38.36 | 82.55 | 82.55 | 85.23 | 100.00 | 100.00 | 38.93 | 44.97 | 53.03 | 45.89 |
| 14: PGAL8A_00028500 | 38.41 | 37.75 | 38.41 | 37.75 | 39.47 | 42.54 | 37.50 | 36.84 | 34.23 | 34.23 | 35.57 | 38.93 | 38.93 | 100.00 | 70.97 | 46.32 | 51.32 |
| 15: PRELSG_1025100 | 39.74 | 39.07 | 39.74 | 39.07 | 39.47 | 42.54 | 37.50 | 38.16 | 40.94 | 40.94 | 40.27 | 44.97 | 44.97 | 70.97 | 100.00 | 52.94 | 52.63 |
| 16: PmUG01_06017500 | 42.42 | 43.94 | 42.42 | 41.67 | 44.78 | 42.54 | 42.54 | 41.79 | 50.76 | 50.76 | 51.52 | 53.03 | 53.03 | 46.32 | 52.94 | 100.00 | 58.96 |
| 17: PocGH01_10034100 | 40.00 | 40.67 | 40.00 | 39.33 | 47.02 | 47.76 | 41.72 | 41.06 | 44.52 | 44.52 | 44.52 | 45.89 | 45.89 | 51.32 | 52.63 | 58.96 | 100.00 |

**Figure S1. Identity matrix (Clustal Omega) calculated from the multiple alignment analysis of *Plasmodium* SUB1-ProM orthologues (Fig. 2a of main manuscript).** The *P. yoelii* 17X (PY17X_1108100) SUB1-ProM primary sequence shows most divergence from the *P. falciparum* sequence, with only 28.67% identity.

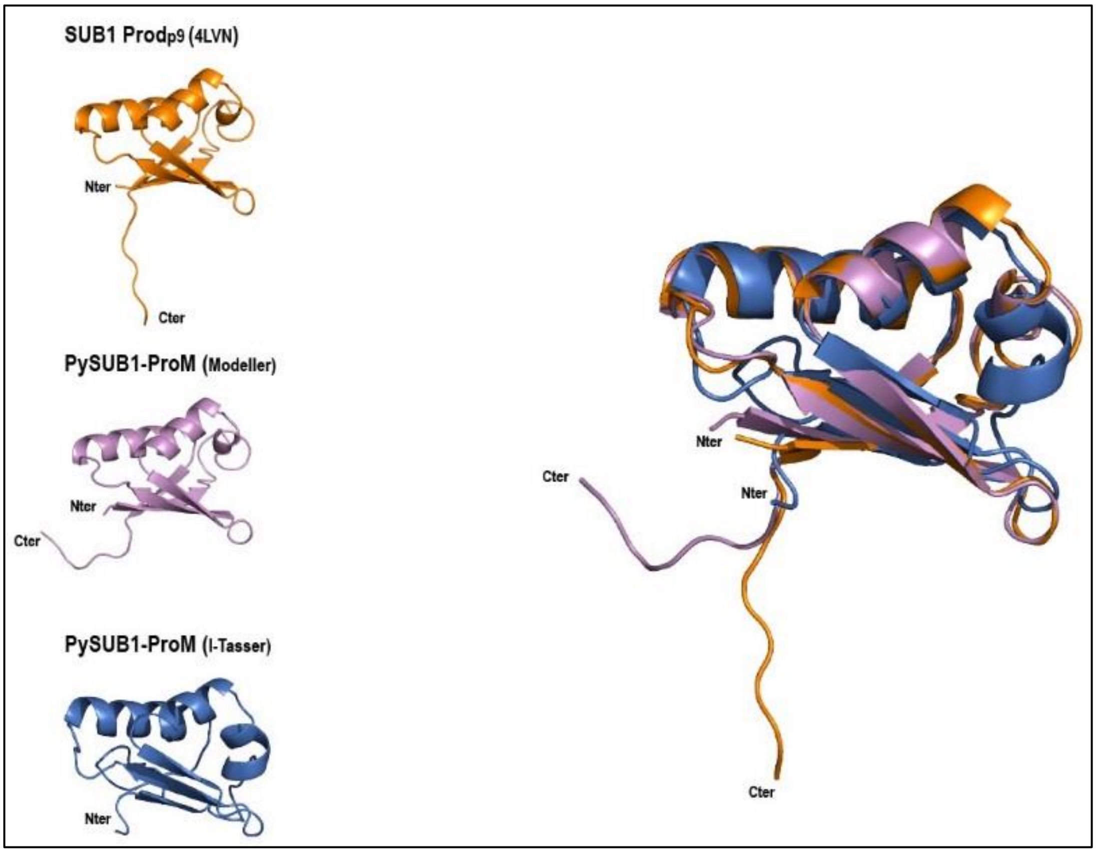


**Figure S2. Conservation of the modelled 3-dimensional structure of *Plasmodium* SUB1-ProM orthologues.** Left-hand side: cartoon representation of SUB1 Prodp9 (orange), shown in isolation from the PfSUB1-Fab complex crystal structure (PDB ID: 4LVN). Below, two partial models of the *P. yoelii* SUB1-ProM (PY17X_1107000), also depicted as cartoons. The first model (Ser72-Leu152; violet) was generated using pGenTHREADER in combination with Modeller, whilst the second (Ser72-Leu152; marine blue) was produced with I-Tasser. Right-hand side: superimposition of the three cartoons to show their spatial agreement. N- and C-termini are indicated.

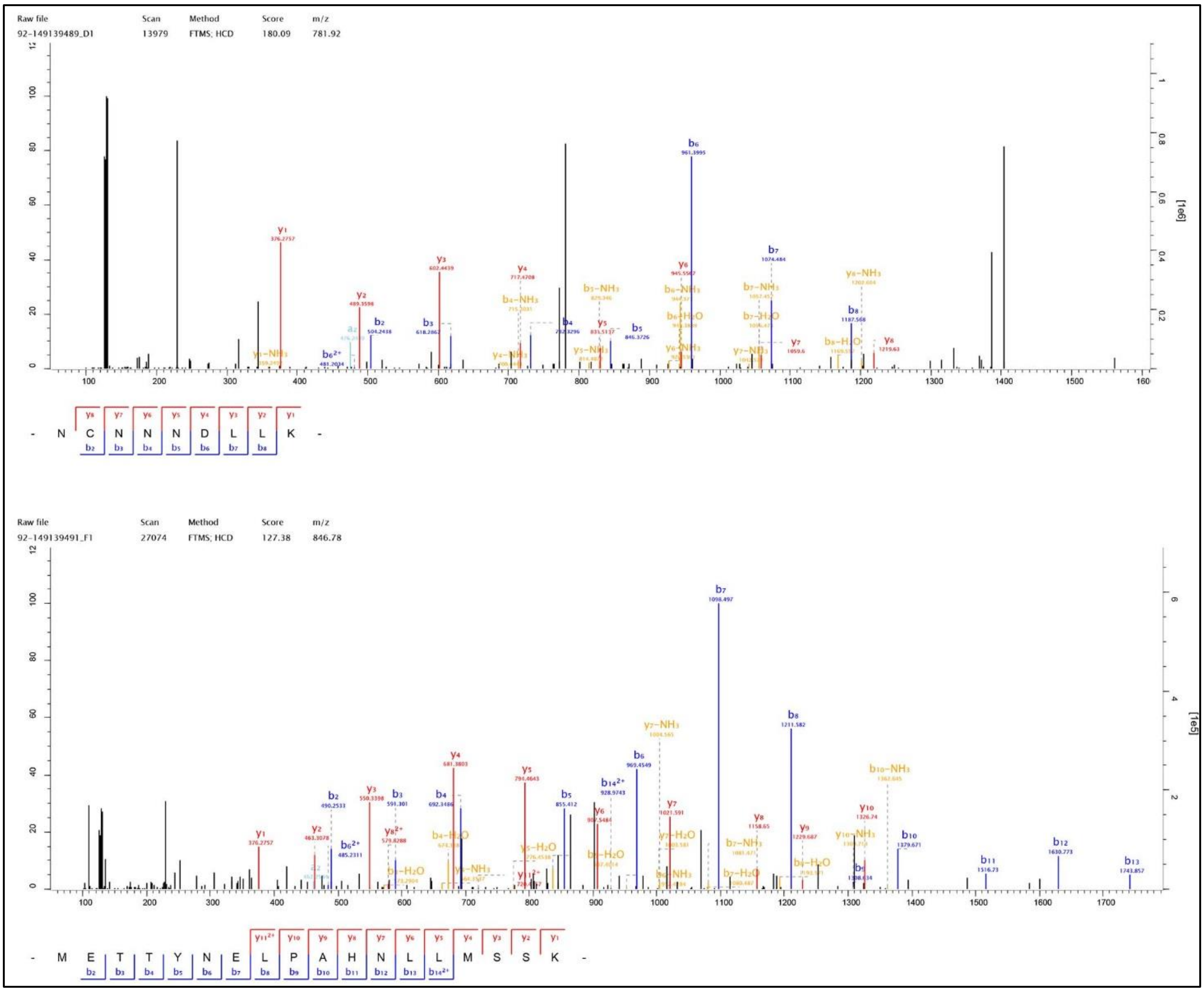


**Figure S3. SUB1-ProM is expressed in asexual blood-stage *P. falciparum* schizonts.** Annotated MaxQuant fragmentation spectra of two SUB1-ProM-derived TMT-labelled tryptic peptides (54NCNNNDLLK62 and 66METTYNELPAHNLLMSSK83) identified in extracts of mature *P. falciparum* 3D7 schizonts. The *m/z* values of selected y and b ions are indicated. Note that *in silico* analysis of the SUB1-ProM sequence showed that complete tryptic digestion was predicted to produce only 6 unique peptides amenable to mass spectrometric identification.

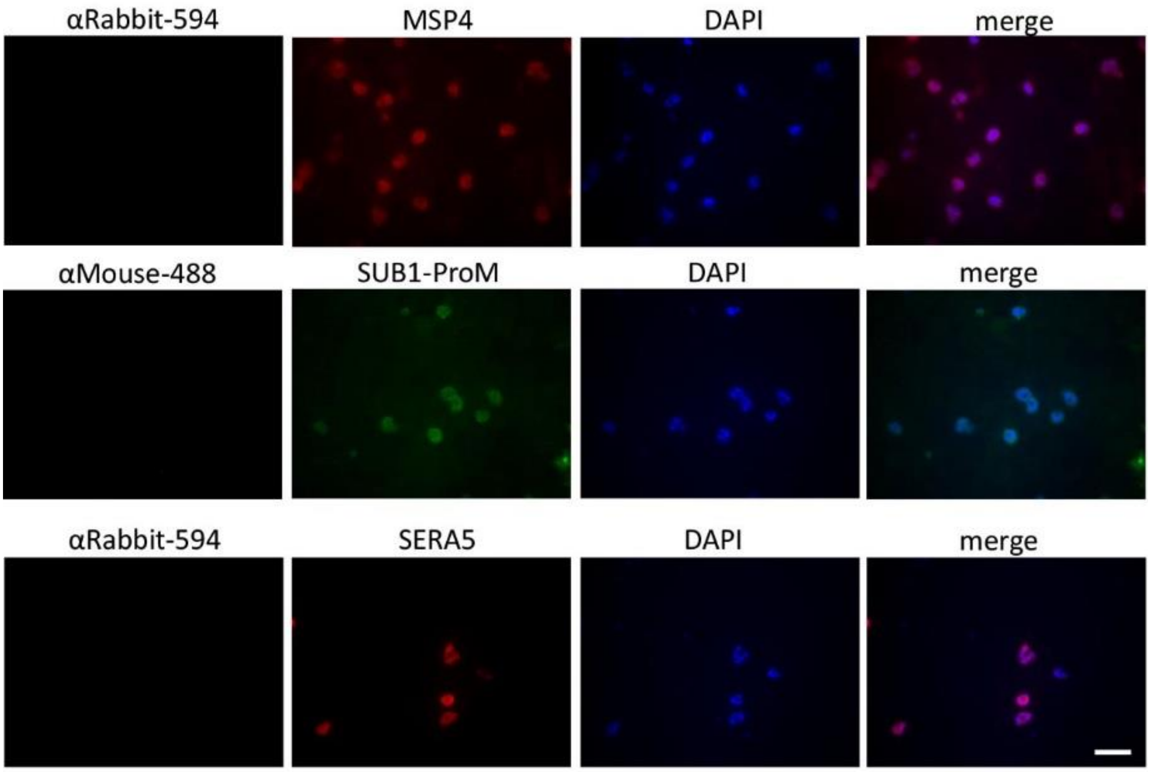


**Figure S4. Immunofluorescence images showing specificity of anti-SUB1-ProM, anti-MSP4 and anti-SERA5 antibodies.** IFA of mature *P. falciparum* 3D7 schizonts probed with the indicated antibodies and imaged at low magnification to display several schizonts per field. The merged images include the nuclear stain DAPI. No signal was obtained with the secondary antibodies alone (left-hand panels). Scale bar, 20 μm.

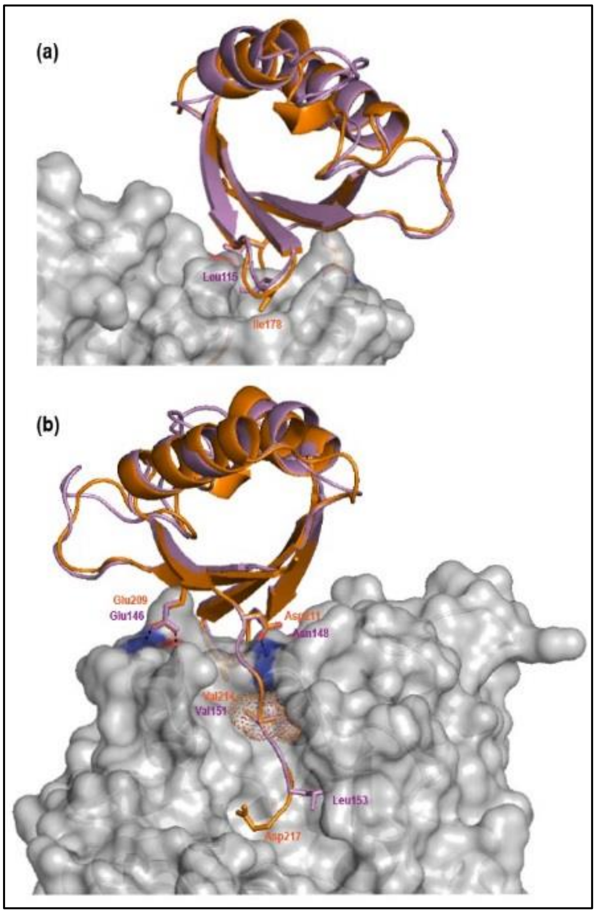


**Figure S5. SUB1-ProM shares conserved features of the PfSUB1 propeptide required for interaction with the PfSUB1 catalytic domain.** (a) Superimposition of the pGenTHREADER/Modeller SUB1-ProM model (violet cartoon) onto the x-ray crystal structure of PfSUB1 Prod$_{p9}$ (orange) complexed to the PfSUB1 catalytic domain (grey molecular surface) (PDB ID: 4LVN). This view faces the back of the active site in order to illustrate a structurally conserved hydrophobic plug involving Prod$_{p9}$ Ile178/SUB1-ProM Leu115 which inserts into a hydrophobic pocket in the PfSUB1 catalytic domain. (b) Rotation of the previous view to face the active site groove, illustrating conservation of the helix capping residues Glu209/Asp211 in Prod$_{p9}$ and Glu146/Asn148 in SUB1-ProM. Hydrogen bond interactions with the PfSUB1 catalytic core are indicated (yellow dotted lines). The extreme C-terminal residues of Prod$_{p9}$ (Asp217) and SUB1-ProM (Leu153) are shown extending into the active site. The side chains of the positionally conserved P4 Val (Prod$_{p9}$ Val214 and SUB1-ProM Val151) are shown as sticks within their dotted surface area, filling the S4 pocket of the PfSUB1 active site cleft. Note that the SUB1-ProM C-terminal tail is one residue shorter than the Prod$_{p9}$ tail and does not fulfil the canonical PfSUB1 active site S2 pocket requirements (Gly or Ala only), indicating a possible non-canonical active site interaction with the PfSUB1 catalytic domain. The SUB1-ProM capping residues (Glu 146, Asn 148) and the SUB1-ProM hydrophobic plug (Leu118) were also structurally conserved as compared to the PfSUB1 propeptide structure 4LVN in both the Robetta and QUARK models of SUB1-ProM.This figure is a replica of Figure 5 in the main text but this time showing the complete model of SUB1-ProM interacting with the catalytic region of PfSUB1.

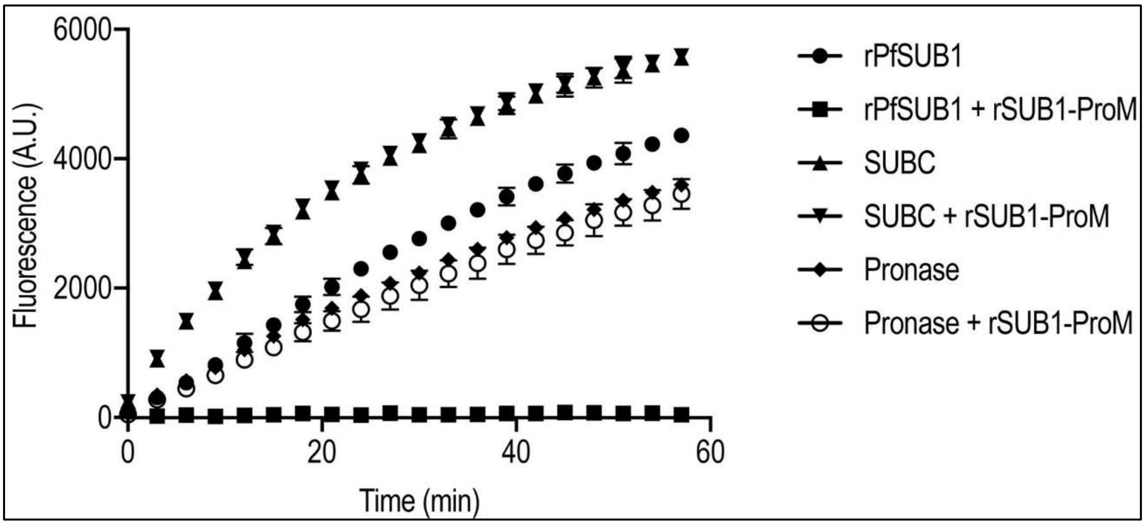


**Figure S6. Recombinant SUB1-ProM is a highly selective inhibitor of PfSUB1 activity.** Progress curves showing the effects of rSUB1-ProM on the proteolytic activity of rPfSUB1, subtilisin Carlsberg (SUBC) and pronase (a commercially available mixture of proteases isolated from the extracellular fluid of *Streptomyces griseus*). Purified rSUB1-ProM (0.5 μM final) was added to: rPfSUB1 (1.14 units/ml) in 25 mM Tris-HCl pH 8.2, 25 mM CHAPS, 12 mM $CaCl_2$; or to SUBC (0.25 ng/ml final) in 20 mM HEPES pH 7.4, 10 mM $CaCl_2$; or to pronase (0.5 μg/ml) in 20 mM Tris-HCl pH 8.2, 150 mM NaCl, such that the final concentration of rSUB1-ProM in each case was 0.5 μM. The rSUB1-ProM-protease mixtures were incubated for 5 min at room temperature before adding the fluorogenic dodecapeptide substrate SERA4st1F-6R12 (0.1 μM final). Increases in fluorescence were monitored over the ensuing 60 min. A protease-alone control (no rSUB1-ProM) for each enzyme was monitored in parallel. All measurements were performed in duplicate and the plotted points indicate the mean average of duplicate measurements. Error bars (±SD) are generally too small to be visible on the plot. Whilst PfSUB1 activity was completely inhibited by 0.5 μM rSUB1-ProM, there was no detectable inhibition of SUBC or pronase activity.

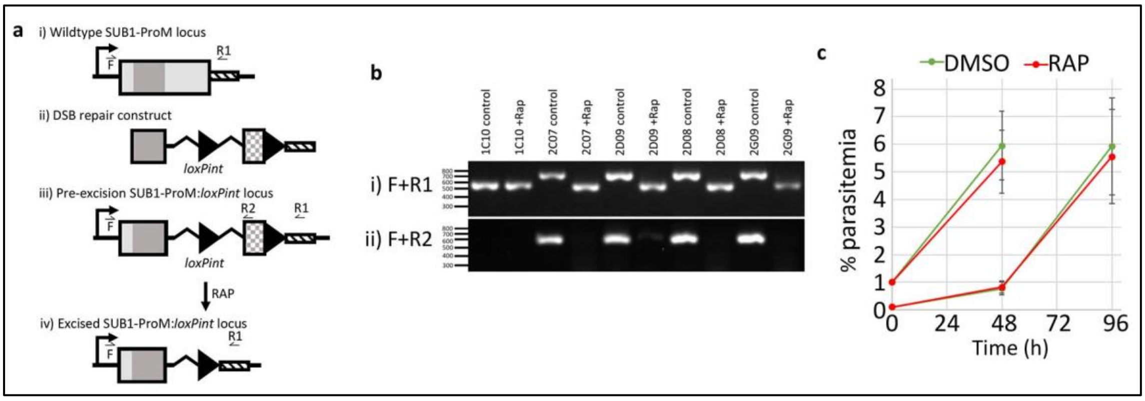


**Figure S7. Conditional genetic modification of the *P. falciparum SUB1-ProM* gene.** (a) Schematics of CRISPR-Cas9 approach used to introduce a *loxP*-intron and 3’ *loxP* site into the 3’ end of the gene encoding SUB1-ProM in a 3D7 DiCre-expressing parasite line. i) wild-type *SUB1-ProM* locus, ii) Cas9 double-strand break repair construct, iii) resultant pre-excision *SUB1-ProM:loxPint* locus and iv) excised *SUB1-ProM:loxPint* locus. Indicated are *loxP* sites (arrow heads), *SERA2* intron sequence (zig-zag) recodonised sequences (chequered), 5’ homology region (dark grey) and 3’ homology region downstream of coding sequence (stripes). (b) PCR fragment sizes confirm excision of the *SUB1-ProM* 3’ region upon rapamycin (RAP) treatment in four *SUB1-ProM:loxPint* parasite clones (2C07, 2D09, 2D08, 2G09) from two independent transfections. Clone 1C10 is a clone of the parental line lacking the *loxPint* integration. i) F+R1 bands demonstrate the size difference across the modified *SUB1-ProM:loxPint* locus upon RAP-induced excision; pre-excision, 706 bp; excised, 545 bp; clone 1C10 control, 569 bp. F+R2 demonstrates the removal of the 3’ region of *SUB1-ProM:loxPint* upon RAP treatment; pre-excision, 551 bp; excised, no product; clone 1C10 control, no product. (c) Truncation of SUB1-ProM did not affect parasite growth over 48 h (for 1% starting-parasitaemia cultures) or 96 h (for 0.1% starting-parasitaemia cultures), as compared to parasites expressing wild-type non-truncated SUB1-ProM. Data show the average parasitaemia values over time of four independent clones. Error bars, ±1 SD.
